# Impact of Nutritional Diet Therapy on Rheumatoid Arthritis Disease Activity

**DOI:** 10.3390/nu18030517

**Published:** 2026-02-03

**Authors:** Elena Deseatnicova, Eugenia Covaliov, Olga Deseatnicova, Rodica Siminiuc, Elena Rezus, Liliana Groppa

**Affiliations:** 1 Department of Rheumatology and Nephrology, State Medical and Pharmaceutical University Nicolae Testemițanu, MD-2004 Chisinau, Moldova; elena.deseatnicova@usmf.md (E.D.); liliana.groppa@usmf.md (L.G.); 2 Department of Food and Nutrition, Faculty of Food Technology, Technical University of Moldova, MD-2004 Chisinau, Moldova; olga.deseatnicova@toap.utm.md (O.D.); rodica.siminiuc@adm.utm.md (R.S.); 3 Department of Rheumatology and Rehabilitation, Grigore T. Popa University of Medicine and Pharmacy, 700115 Iasi, Romania; elena.rezus@umfiasi.ro

**Keywords:** rheumatoid arthritis, diet, inflammation, activity, omega-3 fatty acids, probiotics, dietary fibers

## Abstract

Rheumatoid arthritis (RA) is a chronic autoimmune disease characterized by synovial joint inflammation and different system involvement that results in considerable physical and psychological symptoms. This narrative review investigates the impacts of nutritional diet therapy on RA symptoms, highlighting recent scientific findings in terms of how different dietary components may modulate inflammation and disease activity. Treatment of RA includes conventional and biological disease-modifying antirheumatic drugs (DMARDs) and symptomatic response modifiers, like corticosteroids and non-steroidal antirheumatic drugs (NSAIDS). However, nutritional interventions are becoming more and more popular due to their ability to alter inflammation. The review also focuses on macronutrients such as proteins and fats, stressing the usefulness of omega-3 fat acids/monounsaturated fat acids but warning against high intake of processed carbohydrates/sugars. Besides that, it explores the effects of micronutrients and bioactive compounds like polyphenols which may minimize RA symptoms and result in better disease control together with vitamin D or probiotics. This study highlights that incorporating anti-inflammatory foods can benefit the health and well-being of RA patients. Dietary modification may serve as a supportive approach alongside conventional treatments, helping patients improve both physical and mental aspects of their condition and achieve a better quality of life.

## 1. Introduction

Rheumatoid arthritis (RA) is a chronic systemic autoimmune disorder characterized by symmetric joint inflammation, progressive destruction, and potential extra-articular involvement. Its complex pathogenesis and therapeutic challenges make it a central focus in rheumatology [1,2,3,4]. The onset of RA is influenced by a complex interplay between immune system dysregulation and environmental triggers.

Clinically, RA is characterized by persistent synovial joint inflammation leading to pain, swelling, tenderness, and prolonged morning stiffness, typically lasting more than one hour. As the disease progresses, chronic inflammation may result in cartilage degradation, bone erosion, joint deformities, and functional disability. In addition to articular manifestations, RA frequently presents with systemic features such as fatigue, anemia, osteoporosis, cardiovascular involvement, pulmonary complications, and increased metabolic risk, which collectively contribute to reduced quality of life and increased mortality [1,2,3].

In this review, the term “disease activity” refers primarily to clinical and inflammatory disease activity in rheumatoid arthritis, as commonly assessed using composite indices such as DAS28, inflammatory biomarkers (e.g., CRP, ESR), and clinical manifestations including joint pain and swelling.

From a molecular and immunological perspective, RA is driven by a dysregulated interaction between innate and adaptive immune responses. Activated antigen-presenting cells stimulate autoreactive CD4^+^ T lymphocytes, particularly Th1 and Th17 subsets, leading to excessive production of pro-inflammatory cytokines such as tumor necrosis factor-α (TNF-α), interleukin-6 (IL-6), interleukin-1β (IL-1β), and interleukin-17 (IL-17). These mediators promote synovial hyperplasia, pannus formation, cartilage destruction, and osteoclast-mediated bone resorption. In parallel, B lymphocytes contribute through the production of autoantibodies, including rheumatoid factor (RF) and anti-citrullinated protein antibodies (ACPAs), further amplifying inflammatory cascades and tissue damage [2,3,4].

The standard management of rheumatoid arthritis relies on pharmacological therapy, including conventional and biological disease-modifying antirheumatic drugs, as well as symptomatic agents such as glucocorticoids and non-steroidal anti-inflammatory drugs [5,6]. These treatments remain the cornerstone of disease control and prevention of joint damage. However, despite significant therapeutic advances, many patients continue to experience residual symptoms, comorbidities, or treatment-related adverse effects. This has prompted growing interest in complementary, non-pharmacological strategies, including dietary interventions, as supportive approaches alongside standard medical care.

Several studies in the recent years have been dedicated to understanding the connection between nutrition and prevention or management of RA. However, many of them failed to achieve statistical significance and reproducibility, thus providing weak or controversial evidence in some areas.

The present narrative review wants to highlight various pieces of evidence concerning RA risk factors [7]. Several studies have shown a higher prevalence of RA, in Western societies compared to Eastern and developing countries, suggesting the involvement of some specific environmental triggers [8]. Although genetic susceptibility plays an important role in rheumatoid arthritis development—particularly through HLA-DRB1 alleles encoding the “shared epitope”—genetic factors alone cannot explain the marked differences in RA prevalence observed across populations. Instead, the interaction between genetic background and environmental exposures, including dietary patterns, smoking, gut microbiota composition, and metabolic status, is considered crucial in triggering immune dysregulation and chronic inflammation. This multifactorial framework provides a biological rationale for examining Western dietary habits as modifiable contributors to inflammatory burden and disease activity in RA [1,2,7,8].

The hypothesis is based on the characteristics of Western diets, which are high in the consumption of saturated and trans fats, have a low omega-3 to omega-6 fatty acid ratio, and involve excessive consumption of refined carbohydrates and sugar-sweetened drinks. These factors have been associated with increased inflammatory burden and a higher risk of RA [9,10].

Understanding the role of nutrition and the impact of specific dietary components on inflammatory processes in patients with rheumatoid arthritis is essential for developing evidence-based dietary interventions.

**Figure 1:**
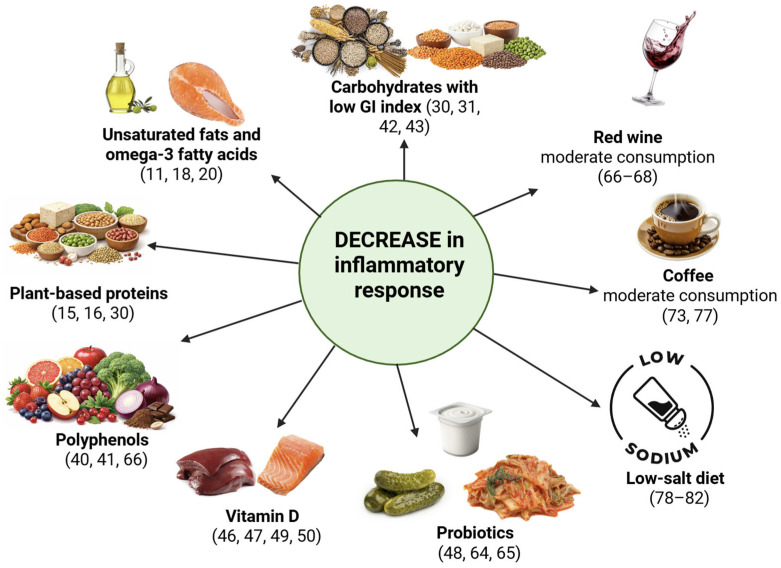
Dietary components associated with a decrease in inflammatory response.

Therefore, it is important to elucidate current perspectives on this topic and to summarize, in a structured manner, which food categories are most commonly recommended as part of nutritional strategies for RA management (see Table 1 and Figure 1).

Table 1 provides a structured overview of the main dietary components and nutrients discussed in this review and summarizes their proposed effects on inflammatory pathways relevant to rheumatoid arthritis. The table is intended as a conceptual synthesis, integrating mechanistic, observational, and interventional evidence. Importantly, the entries differ in the level of supporting evidence and should be interpreted as indicative of biological plausibility rather than as a hierarchy of clinical effectiveness.

As summarized in Table 1, the strength of evidence varies across dietary components, with some associations supported mainly by mechanistic data and others by human interventional studies.

Figure 1 shows a conceptual schematic illustrating proposed mechanisms by which dietary components may modulate inflammatory pathways relevant to rheumatoid arthritis. The pathways shown are based largely on mechanistic and preclinical evidence and should not be interpreted as direct indicators of clinical efficacy or therapeutic benefit.

As illustrated in Figure 1, multiple dietary components may contribute to the modulation of inflammatory pathways in rheumatoid arthritis through complementary mechanisms. Importantly, the strength and type of evidence supporting these associations vary, ranging from well-established clinical trial data (e.g., omega-3 fatty acids) to findings derived mainly from mechanistic and observational studies.

## 2. Materials and Methods

A narrative review approach was employed to explore the relationship between nutritional diet therapy and RA activity. The literature search was carried out in the main scientific databases, including PubMed, ScienceDirect, Google Scholar, and Web of Science, covering publications between 2010 and 2025.

The search strategy combined the following keywords and their variants: “*rheumatoid arthritis*”, “*diet*”, “*nutrition*”, “*omega-3 fatty acids*”, “*vitamin D*”, “*polyphenols*”, “*probiotics*”, “*dietary fiber*”, and “*inflammation*”. Reference lists of relevant articles were also manually screened to identify additional pertinent studies.

The initial search yielded approximately 420 records. After removal of duplicates and screening of titles and abstracts, about 170 articles were selected for full-text assessment. Ultimately, 82 publications were included in the final review.

Inclusion criteria comprised peer-reviewed clinical trials, observational studies, systematic reviews, and meta-analyses that examined the relationship between dietary patterns, specific nutrients, or bioactive food components and the incidence, activity, or clinical outcomes of rheumatoid arthritis. Studies focusing on both macronutrients and micronutrients, as well as gut microbiota-related dietary interventions, were considered.

Exclusion criteria included conference abstracts without full text, editorials, commentaries, case reports, non-peer-reviewed literature, and studies that did not directly address nutritional aspects of rheumatoid arthritis or lacked sufficient methodological detail.

Articles published in English were primarily considered. In addition, publications in Romanian, French, and Russian were included when an English abstract was available and the full text could be reliably assessed by the authors.

Although no formal systematic review protocol was applied, all included studies underwent careful screening at the title, abstract, and full-text level to ensure relevance and scientific quality.

## 3. The Role of Diet and Macronutrient Consumption in RA

Healthcare providers often receive questions about dietary recommendations from patients who have rheumatoid arthritis because they observe that certain foods can help or worsen their symptoms [32,33]. For example, some of the popular mentioned items are red meat, alcohol, and soft drinks which intensify symptoms, while fish and berries may ameliorate them [6].

The evidence discussed below includes mechanistic studies (in vitro, animal models, immunological or molecular pathways), observational human studies (cross-sectional, cohort, case–control studies) and interventional trials (randomized controlled trials, dietary interventions, supplementation trials). Where available, human interventional evidence is highlighted, while mechanistic and observational findings are interpreted as supportive but indirect. Studies suggest that certain dietary components—such as omega-3 fatty acids, probiotics, vitamin D, and antioxidants—may influence inflammatory processes and selected clinical outcomes in RA, although the level of evidence varies among nutrients. Omega-3s have been linked to lower ESR and fewer swollen or tender joints, while probiotics may help control disease activity. Evidence regarding vitamin D’s ability to reduce disease activity is limited; however, antioxidants and bioactive compounds offer potential in reducing symptoms and improving disease activity [34,35]. Vadell et al. (2020) [34] conducted a single-blinded crossover trial including 50 patients with RA who were randomly assigned to an intervention diet containing a portfolio of suggested anti-inflammatory foods or a control diet similar to the general Swedish diet, each for a period of 10 weeks with a 4-month washout before crossover. The study did not demonstrate significant or clinically meaningful reductions in DAS28 or its individual components. The relatively small sample size, short intervention duration, and potential carry-over effects represent important limitations that may have reduced the statistical power to detect clinically relevant changes [34].

### 3.1. Protein Intake

The relationship between dietary protein intake and RA activity remains a subject of ongoing research and appears to depend significantly on the source of protein (animal vs. plant), its impact on inflammatory pathways, gut microbiota, and muscle mass maintenance. Evidence regarding protein intake and RA activity derives primarily from mechanistic studies and observational human research, with limited interventional evidence available. Red meat contains high levels of saturated fats, advanced glycation end products, and heme iron, all of which may activate proinflammatory signaling pathways, including NF-κB and Toll-like receptor 4 mediated responses. Increased C-reactive protein and IL-6 levels have been observed in individuals with diets high in red meat, which are biomarkers of disease activity in RA [36]. Jin et al. (2021) [11] conducted a cross-sectional study at Peking University People’s Hospital, recruiting 733 RA patients diagnosed according to ACR criteria, of which 707 were included after exclusions. Dietary intake prior to symptom onset was assessed with a rheumatologist-assisted questionnaire. Higher red meat consumption was linked to earlier RA onset, particularly among smokers and overweight participants. However, the single-center design, recall bias, and the cross-sectional nature of the study limit causal interpretation [11]. Moreover, a systematic review and meta-analysis reported a moderate association between high red meat intake and increased RA risk [12]. Meat proteins lead to changes in the gut microbiota, favoring protein-fermenting bacteria that produce potentially proinflammatory metabolites such as ammonia, hydrogen sulfide, and phenolic compounds. These metabolites can disrupt intestinal barrier function and promote systemic immune activation, possibly exacerbating autoimmune processes in RA [14].

Plant-based proteins are typically low in saturated fat and high in anti-inflammatory phytochemicals, fiber, and polyunsaturated fatty acids, which may exert a protective effect. Diets rich in plant proteins are associated with lower inflammatory biomarkers and, in some studies, improved DAS28 scores in RA patients [15]. Plant protein intake also supports fiber-fermenting bacteria, leading to the production of short-chain fatty acids, like butyrate, which help to maintain intestinal barrier integrity and modulate immune response [17]. On the other hand, a prospective study by Hagen et al. found no clear association between total protein intake and risk of developing RA, but higher red meat intake was weakly associated with increased RA risk in some subgroups [13]. Some intervention studies show that vegan and Mediterranean diets, which are lower in animal protein and richer in plant sources, are associated with reduced RA activity [16]. To date, interventional trials directly manipulating animal protein intake in RA patients remain scarce, limiting causal inference.

### 3.2. Fat Intake

#### 3.2.1. Unsaturated Fats

Several studies suggest that omega-3 fatty acids, including eicosapentaenoic acid and docosahexaenoic acid, have demonstrated robust anti-inflammatory effects, supported by mechanistic data and clinical trials, relevant to RA pathogenesis. These fatty acids competitively inhibit the metabolism of arachidonic acid by cyclooxygenase and lipoxygenase, leading to a reduction in pro-inflammatory eicosanoids such as prostaglandin E2 and leukotriene B4 [18]. They may help alleviate certain symptoms of rheumatoid arthritis, potentially due to their anti-inflammatory properties [11,15,17]. A study by Rosillo et al. (2016) described that extra virgin olive oil had a potent effect on arthritis prevention when comparing a diet containing extra virgin olive oil in a murine model of collagen-induced arthritis (CIA) where it depicted less joint swelling and cartilage erosion as compared to other oils [19].

#### 3.2.2. Monounsaturated Fatty Acids (MUFA)

Taking into account the features of the Mediterranean diet regarding the fatty acid profile of fats, including olive oil and fish, the investigation carried out by Matsumoto et al. (2017) proved that the intake of MUFAs may decrease disease activity [37]. Brzeski et al. (1991) and Linos et al. (1991) also came up with similar results concerning the effects of olive oil in suppressing RA-related symptoms and linking it to lesser risk of RA in the general population consuming olive oil [38,39].

#### 3.2.3. Polyunsaturated Fatty Acids (PUFA)

The meta-analysis of Gioxari et al. (2018) [20] established that oral supplementation of omega-3 fatty acids can effectively improve indicators of disease severity such as tender joint count and ESR, along with pain scores, quality of life, and glucocorticoid dosage. This analysis included 20 RCTs with 717 RA patients in intervention and 535 in control groups, with a minimum intervention period of 3 months. However, heterogeneity across trial protocols and a moderate risk of bias were noted, which limit the strength and generalizability of the conclusions [20]. On the other hand, there are some publications showing that omega-6 and omega 9 fatty acids in certain conditions may have proinflammatory effects. When omega-6 intake is excessive and omega-3 intake is low, metabolism favors arachidonic acid-derived pro-inflammatory eicosanoids, while if omega-6 intake includes sufficient gamma-linolenic acid and Dihomo-γ-linolenic acid, or is balanced with omega-3s, anti-inflammatory pathways may dominate [21,22,23,24,25].

#### 3.2.4. Saturated Fats

According to current evidence, palmitic acid and stearic acid fatty acids can activate Toll-like receptor 4, subsequently triggering key intracellular signaling pathways such as NF-κB and MAPK. This activation promotes the expression of pro-inflammatory cytokines [26].

Additionally, those acids induce endoplasmic reticulum stress and the generation of reactive oxygen species, activating the NLRP3 inflammasome. The activation of this multiprotein complex further enhances IL-1β secretion, thereby amplifying systemic inflammation [27].

Taken together, these mechanisms suggest that a diet high in saturated fats may contribute to the persistence and severity of inflammation in RA. These findings are consistent with current dietary recommendations advocating for a reduction in saturated fat intake as part of anti-inflammatory nutritional strategies.

### 3.3. Carbohydrate Intake

Glycemic index (GI) again plays a role on the effect carbohydrates have in the body, and in this case, the subjects observed an effect on inflammation. GI explains the quick rise in blood glucose and insulin levels triggering a rise in the production of pro-inflammatory cytokines [29]. Some other investigations showed that the increased consumption of refined carbohydrates and sugars leads to the increase in inflammatory markers [18,28]. On the other hand, carbohydrate foods classified as having a low GI that provide fibers such as whole grains, vegetables, and legumes were found to reduce inflammation and could be beneficial to RA-affected persons [30]. A review by Gerontiti et al. (2024) demonstrated that low-GI diets help to minimize glucose fluctuations and are associated with lower systemic inflammation—noted by reductions in markers like c-reactive protein [31].

## 4. The Role of Diet and Micronutrient and Biological Active Compounds Consumption in RA

The scientific literature review also included several works that showed the importance of specific micronutrients in RA symptom reduction and better condition control [20,21,22,23,39]. Compounds such as Vitamin D, polyphenols, probiotics, and others help in suppression of inflammation and immune regulation in rheumatoid arthritis. It is suggested that these nutrients may be useful in altering the pathogenesis of the disease by decreasing oxidant activity and replenishing depleted nutrient pools.

### 4.1. Polyphenols

Evidence supporting the role of polyphenols in rheumatoid arthritis derives primarily from mechanistic studies and observational data, with a limited number of interventional trials. Polyphenols help to alleviate rheumatoid arthritis through modulating a large number of genes relating to RA such as MAPK, IL-1β, IL-6, TNF-α, NF-κB, JNK, ERK 1/2, AP-1, and COX-2 [40]. Many researchers have investigated polyphenols concerning their antirheumatic impact. Rosillo et al. (2014), who used polyphenol extract in CIA mice, also recorded reduced expression in IL-1β, TNF-α, PGE2, JNK, 1κB-α, and p-65 [41]. Yoon et al. (2013), Decendit et al. (2013), Umar et al. (2013), and other authors [42,43,44] also found similar results. The published works they cited described the performance of hesperidin, kaempferol, resveratrol, and others on their bodies as positive. Cocoa polyphenols affect both the system’s innate and adaptive immunity, stimulating the response. An anti-arthritic effect of cocoa flavanols has been reported in arthritis models, and feeding cocoa flavanols offered partial protection by suppression of autoantibodies, oxidative stress, and reactive oxygen species (ROS) levels and further by preventing T-reg serum levels from falling, which, however, does not provide substantial inhibition of arthritis, as evidenced by joint swelling [22,23].

### 4.2. Vitamin D

The evidence regarding vitamin D in rheumatoid arthritis includes mechanistic insights, observational associations, and several interventional studies, although results from randomized trials remain heterogeneous. Vitamin D has been identified as a possible adjuvant therapy for rheumatoid arthritis for many years [45,46,47]. It is synthesized in the skin when exposed to UVB or obtained from nutrition. Calcitriol bounds to its ligand vitamin D receptor (VDR) which is located on the immune system cells. Reports indicate vitamin D deficiency is present in 30% to 63% of the population. Vitamin D is important for immunity as it participates in the regulation of many parts of the immune system. It suppresses Th1 and Th17 and favors Th2 and Treg, resulting in reduced T-cell effector immune responses [48]. This may explain why its deficiency leads to increased concentration of pro-inflammatory cytokines in the blood of the patients with RA. Research also implies that vitamin D has a possibility of regulation of the immune tolerance, halting the initiation and development of inflammatory arthritis such as RA. Kerr et al. (2011) [49] reported high rates of vitamin D insufficiency in RA, with 850 US veterans studied; insufficiency (<30 ng/mL) was found in 84% and deficiency (<20 ng/mL) in 42% of cases. Deficiency correlated with higher tender joint counts and hs-CRP, though the cross-sectional design and specific veteran cohort limit generalizability [49]. Furthermore, reduced levels of vitamin D have been reported in other autoimmune diseases, apart from RA, further proving the way it influences immune cell functions and their differentiation. Some clinical investigations demonstrated negative correlation between vitamin D and the activity of the RA. For example, research showed that the patients with active RA had a significantly lower level of vitamin D compared with the patients with RA in remission [50]. This brings out the point that optimal vitamin D status may play a supportive role in the management of RA, which may well be the case for other autoimmune diseases [29,51]. However, interventional trials evaluating vitamin D supplementation have produced heterogeneous results, with no consistent reduction in disease activity across studies.

### 4.3. Trace Elements (Iron, Zinc, and Copper)

Important trace elements such as iron, zinc, and copper are instrumental in the regulation of immune function and antioxidant defense, as well as in maintaining tissue homeostasis, all critical in the pathophysiology of rheumatoid arthritis (RA). Chronic systemic inflammation in patients with RA is often accompanied by marked changes in trace element distribution, which may be not only an indication of dietary intake, but also the effect of inflammation on absorption, transport, storage, and utilization. As a result, dietary therapy in RA should be considered mainly as a supportive strategy aimed at maintaining mineral homeostasis rather than as a direct means of correcting serum deficiencies.

Zinc is currently one of the most studied trace elements in RA owing to its central role in both adaptive and innate immune responses. Numerous clinical studies have reported low serum zinc concentrations in patients with RA compared to healthy controls, an observation that may be related to active inflammation and oxidative stress. Pro-inflammatory cytokines might mediate hepatic sequestration of zinc by synthesis of metallothionein, which results in decreased serum levels of zinc even after adequate diet. Zinc deficiency has even been associated with inhibited activity of antioxidant enzymes, increased production of reactive oxygen species, and enhanced release of pro-inflammatory mediators, conditions that can aggravate synovial inflammation and joint damage [52,53]. Certain clinical and laboratory parameters have been reported to benefit from zinc supplementation or a diet rich in zinc, although its influence may be indirect by targeting immune and redox pathways, not direct zinc concentration normalization.

Conversely, elevated circulating copper levels are frequently found in RA, owing to stimulation of the synthesis of ceruloplasmin, which is an acute-phase protein and is higher in individuals with RA during inflammation. Increased copper was positively correlated with inflammatory biomarkers and disease activity scoring, indicating that copper status accounts for a more inflammatory burden than dietary excess. As a result, dietary therapy should avoid excessive copper intake and instead emphasize balanced, anti-inflammatory dietary patterns [54,55].

In RA, iron metabolism is also significantly altered with diminished circulating iron levels and increased concentrations within inflamed synovial tissue, the latter resulting in anemia of chronic disease. Studies have documented a negative association between circulating iron levels and inflammatory markers and disease activity, thus stressing the role of inflammation control in iron homeostasis restoration. Although iron as dietary source is necessary, bioavailability in RA is mainly an outcome of inflammation regulation, not only intake [56,57].

Thus, the evidence so far suggests that changes in iron, zinc, and copper status in RA are generally secondary to chronic inflammation. It is possible that dietary treatment can nevertheless play an important role in maintaining mineral homeostasis and preventing oxidative stress, but it should be considered a component of a more comprehensive anti-inflammatory program and not a point of specific mineral therapy alone. This interpretation is corroborated by recent Mendelian randomization studies that have not shown a causal link between genetically predetermined mineral concentration and RA risk level [58]

### 4.4. Probiotics

There is an evidence from human clinical research that RA patients have distinct measures of gut microbiota and the dysbiosis may trigger the development of inflammatory arthritis [59,60,61,62]. The relationship between probiotics and RA has been explored primarily through mechanistic studies and small-scale interventional trials. There is a positive impact of probiotics on human health, especially since they help decrease inflammation caused by bacteria, which is capable of altering the intestinal barrier and therefore causing extra antigenic stimulation [63]. Such studies reveal that there is a body of literature showing that an intervention with probiotics has been associated with modest improvements in DAS28 in some clinical trials [64,65]. Nevertheless, the meta-analysis conducted by Aqaeinezhad Rudbane et al. (2018) evince that the role of probiotics appears slightly less important in RA management, which calls for future research in this respect [59].

### 4.5. Alcohol

Influence of alcohol consumption on people with rheumatoid arthritis (RA) is still an issue that is being investigated. Some publications suggested that especially moderate use of alcohol might even be helpful to the condition [66,67,68]. It is also well documented that alcohol consumption can make the symptoms worse and lead to poorer prognosis [69]. Appropriate intake of alcohol, specifically beer, has been believed to be linked with rheumatoid arthritis. A paper in the “Annals of the Rheumatic Diseases” revealed that moderate alcohol consumption lowered the risk of RA in women [66,70]. Studies have said that the ability of alcohol to dampen inflammation in the body may be responsible for this reduced risk, depending, of course, on the type of alcohol that is being consumed, the primary example being polyphenols, such as resveratrol, an ingredient of wines.

On the other hand, high alcohol consumption is considered to have negative effects on anyone with RA. Alcohol constitutes a risk factor when used in moderate or excessive quantities with drugs used to treat RA, including methotrexate and NSAIDs. Such drugs may lead to enhanced liver toxicity and gastrointestinal complications [71]. Moreover, drinking leads to a damaged immune function which may worsen the inflammation levels in RA [72].

### 4.6. Caffeine

Moderate association between the amount of coffee intake and seropositive RA risk has been reported previously [73]. As we currently know, the debate on the interaction between coffee or caffeine and rheumatoid arthritis is still ongoing. Coffee is one of the most widely liked beverages taken by many people, has numerous health benefits, and at the same time takes a middle position in the development and severity of RA, depending on many conditions such as genetics, the amount of coffee consumed, and others. Caffeine, the main alkaloid consumed by humans through coffee, has been established to have anti-inflammatory effects. According to several studies, it was pointed out that intake of caffeine can reduce the inflammatory response observed in RA through neutralization of cytokines [74,75]. Moreover, moderate coffee intake has shown to decrease the chance of RA because of its antioxidant content which can alleviate oxidative stress responsible for the development of chronic inflammatory diseases such as RA [76].

However, there are certain undesirable effects identified towards coffee and caffeine consumption that have been pointed out by some researchers. Asoudeh et al.’s (2022) [77] meta-analysis proves that high ingestion of coffee could increase risks of seropositive RA. The exact nature of this risk’s mechanism is unknown but may entail comprehensive interfaces between caffeine and immune systems [77].

### 4.7. Salt

High salt consumption has been implicated in the modulation of immune responses and may influence the pathogenesis and activity of autoimmune diseases, including RA. Sodium chloride enhances the expression of serum- and glucocorticoid-regulated kinase 1, which facilitates Th17 polarization via the IL-23/IL-23R signaling axis [78,79]. High salt intake has been shown to promote differentiation of naive CD4^+^ T cells into Th17 cells, producing IL17, which plays a crucial role in RA by contributing to synovial inflammation, osteoclastogenesis, and cartilage destruction. Sodium overload can also potentiate macrophage activation, leading to increased secretion of proinflammatory cytokines such as TNF-α, IL-6, and IL-1β, central mediators of RA pathogenesis [80]. High salt intake can disrupt the composition of the gut microbiota, leading to dysbiosis and increased intestinal permeability that results in translocation of microbial components into the systemic circulation. This contributes to systemic low-grade inflammation and may act as a trigger for autoimmune responses [81]. A Spanish cohort study found that higher sodium intake was associated with greater DAS28 and reduced response to DMARDs, particularly in smokers [82].

## 5. Clinical Implications and Practical Considerations

From a clinical perspective, the evidence reviewed in this article indicates that dietary interventions in rheumatoid arthritis differ substantially in both type and strength of supporting evidence, as summarized in Table 2.

Interventions supported by human interventional trials and meta-analyses may be considered as reasonable adjuncts to standard pharmacological therapy, whereas others should currently be regarded as supportive or exploratory.

Among the dietary approaches discussed, omega-3 fatty acid supplementation and Mediterranean-style dietary patterns are supported by the most consistent interventional evidence, demonstrating modest but clinically meaningful improvements in inflammatory markers, disease activity scores, and symptom burden. These interventions may therefore be considered in routine clinical counseling for patients with rheumatoid arthritis.

Other factors, including vitamin D optimization, probiotic supplementation, and increased intake of polyphenol-rich foods, are supported primarily by mechanistic and observational evidence, with interventional findings remaining heterogeneous. While these approaches appear biologically plausible and safe, they should currently be interpreted as complementary strategies rather than evidence-based therapeutic interventions.

Finally, dietary components such as reduced red meat intake, limitation of refined carbohydrates, and moderation of alcohol consumption are supported mainly by observational and mechanistic data. These measures align with general anti-inflammatory and cardiometabolic health recommendations and may be reasonably advised, although direct evidence for RA-specific clinical benefit remains limited.

While the mechanistic pathways illustrated in Figure 1 provide biological plausibility for many dietary factors, translation into clinically meaningful benefit depends on human interventional evidence, which remains variable across dietary components (Table 2).

Overall, dietary interventions should be individualized, integrated with pharmacological management, and framed as supportive measures rather than substitutes for disease-modifying therapy.

## 6. Conclusions

As this article has brought out, nutritional diet therapy represents a promising complementary approach that may contribute to symptom management and potentially to risk reduction in RA. Modern studies show that certain RA symptoms may greatly depend on the kind of food that is consumed. Eating habits in western civilizations that are rich in fats, specifically saturated and *trans* fats, refined carbohydrates, sugar sweetened beverages, and red meat may influence higher prevalence of inflammation related diseases and RA. On the other hand, diets with omega-3 fatty acids, polyphenols, and other anti-inflammatory nutrients are considered useful for modulating inflammatory pathways and supporting disease management, with the strongest clinical evidence available for omega-3 fatty acids.

Of the macronutrient constituents of food, proteins, especially of fish and plant origin, appear to be anti-inflammatory. Supplementation with omega-3 polyunsaturated fatty acids, particularly those derived from marine sources such as fish oil, has been consistently associated with a reduction in inflammatory biomarkers and a measurable decrease in disease activity in patients with rheumatoid arthritis. Olive oil, an important component of Mediterranean diet, has also demonstrated anti-inflammatory properties. The positive effects of micronutrients like vitamin D and polyphenols extend to the utilization of a nutrient-dense diet for persons with RA.

Emerging evidence indicates that probiotic supplementation may beneficially modulate the composition and activity of the gut microbiota, contributing to the stabilization of the intestinal barrier and attenuation of systemic inflammation in RA. These interactions emphasize the necessity of taking dietary methods as a part of RA management along with traditional approaches.

## Figures and Tables

**Table 1:** The influence of the main nutrients on inflammatory response.

	Contain	Action	Effect
Proteins			
Red meat	High levels of saturated fats, advanced glycation end products, and heme iron	Activate proinflammatory signaling pathways, including NF-κB and Toll-like receptor 4 mediated responses [11,12,13]	Proinflammatory
	Stimulates protein-fermenting bacteria that produce ammonia, hydrogen sulfide, and phenolic compounds	Disrupt intestinal barrier function and promote systemic immune activation [14]	Proinflammatory
Plant based proteins	Low in saturated fat and high in anti-inflammatory phytochemicals (ex. genistein, daidzein, glycitein, soyasaponins, and phytic acid), fibers, and polyunsaturated fatty acids	Inhibit NF-κB activation, reduce TNF-α, IL-6 production, suppress COX-2 [15,16]	Anti-inflammatory
	Fibers that support fiber-fermenting bacteria	Production of short-chain fatty acids, like butyrate, which maintain intestinal barrier integrity and modulate immune response [17]	Anti-inflammatory
Fats			
Monounsaturated fatty acids (*n*-3)	Eicosapentaenoic acid, docosahexaenoic acid, and α-linolenic acid	Replace arachidonic acid in membranes, reducing the substrate available for pro-inflammatory eicosanoid synthesis. Precursors for resolvins, protectins, and maresins specialized pro-resolving anti-inflammatory lipid mediators Inhibit NF-κB activation, reduce TNF-α, IL-6 production [11,15,17,18,19]	Anti-inflammatory
Polyunsaturated fatty acids (*n*-6)	Linoleic acid, arachidonic acid, and product of their peroxidation generated during frying (hydroxynonenal)	Precursors for pro-inflammatory eicosanoids, including Prostaglandin E2, Thromboxane A2, Leukotriene B4 [20] May become target for reactive oxygen spices even after incorporation in membranes [21]	Pro-inflammatory
	Dihomo-γ-linolenic acid γ-linolenic acid Linoleic acid when balanced with omega 3	Precursor to anti-inflammatory eicosanoids such as: prostaglandin E1 and 15-hydroxyeicosatrienoic acid [21,22,23,24,25]	Neutral or mild -antiinflammatory when balanced with omega-3
Poliunsaturated fatty acids (*n*-9)	Oleic acid	Inhibit NF-κB activation, reduce TNF-α, IL-6 production [24]	Neutral or mildly anti-inflammatory Proinflammatory in combination with saturated fats
Saturated fats	Palmitic acid Stearic acid	Activate Toll-like receptor 4, NF-κB and MAPK pathways, promoting expression of IL-6, TNF-α, IL-1β. ER stress and ROS, triggering the NLRP3 inflammasome, enhancing IL-1β release [26,27]	Pro-inflammatory
Carbohydrates			
With high glycemic index	Refined carbohydrates and sugars	Contributes to insulin release and production of proinflammatory cytokines [18,28,29]	Pro-inflammatory
With low glycemic index	Low glycemic index classified sugars	Gradual rise in blood sugar and insulin levels what reduces oxidative stress, endothelial activation, and inflammatory signaling [30,31]	Anti-inflammatory

**Table 2:** Type and level of evidence supporting dietary factors in rheumatoid arthritis.

Dietary Factor/Pattern	Mechanistic Evidence	Observational Human Evidence	Interventional Human Evidence	Overall Interpretation
Omega-3 fatty acids	Strong (NF-κB, eicosanoids, resolvins)	Cohort and case–control	RCTs and meta-analyses	Moderate–strong evidence for symptom reduction
Red meat	Gut dysbiosis, TLR4 activation	Association with RA risk	Limited	Suggestive harmful association
Mediterranean diet	Anti-inflammatory pathways	Cohort studies	Small RCTs	Promising dietary pattern
Vitamin D	Immune modulation (Th17/Treg)	Deficiency–activity correlation	Mixed RCT results *	Supportive but inconclusive
Probiotics	Gut–immune axis	Dysbiosis studies	Small RCTs, mixed meta-analyses *	Emerging evidence
Polyphenols	Strong animal and in vitro	Limited **	Mostly preclinical *	Mechanistic support only

*—limited evidence, **—lack of evidence.

## Data Availability

No new data were created or analyzed in this study. Data sharing is not applicable to this article.
